# Modafinil-Induced Changes in Functional Connectivity in the Cortex and Cerebellum of Healthy Elderly Subjects

**DOI:** 10.3389/fnagi.2017.00085

**Published:** 2017-03-30

**Authors:** Miriam Punzi, Tommaso Gili, Laura Petrosini, Carlo Caltagirone, Gianfranco Spalletta, Stefano L. Sensi

**Affiliations:** ^1^Department of Neurosciences, Imaging and Clinical Sciences, “G. d’Annunzio” University of Chieti-PescaraChieti, Italy; ^2^Molecular Neurology Unit, Center of Excellence on Aging and Translational Medicine (Ce.S.I.-Me.T.), “G. d’Annunzio” University of Chieti-PescaraChieti, Italy; ^3^Museo Storico della Fisica e Centro Studi e Ricerche Enrico FermiRome, Italy; ^4^Santa Lucia FoundationRome, Italy; ^5^Department of Psychology, Section of Neuroscience and “Daniel Bovet” Neurobiology Research Center, Sapienza University of RomeRome, Italy; ^6^Department of Medicine of Systems, University of Rome Tor VergataRome, Italy; ^7^Departments of Neurology and Pharmacology, Institute for Mind Impairments and Neurological Disorders, University of California, Irvine, IrvineCA, USA

**Keywords:** cognitive enhancing drugs, dopamine, aging, resting state fMRI, connectivity, graph theory, eigenvector centrality

## Abstract

In the past few years, cognitive enhancing drugs (CEDs) have gained growing interest and the focus of investigations aimed at exploring their use to potentiate the cognitive performances of healthy individuals. Most of this exploratory CED-related research has been performed on young adults. However, CEDs may also help to maintain optimal brain functioning or compensate for subtle and or subclinical deficits associated with brain aging or early-stage dementia. In this study, we assessed effects on resting state brain activity in a group of healthy elderly subjects undergoing acute administration of modafinil, a wakefulness-promoting agent. To that aim, participants (*n* = 24) were investigated with resting state functional Magnetic Resonance Imaging (rs-fMRI) before and after the administration of a single dose (100 mg) of modafinil. Effects were compared to age and size-matched placebo group. Rs-fMRI effects were assessed, employing a graph-based approach and Eigenvector Centrality (EC) analysis, by taking in account topological changes occurring in functional brain networks. The main finding of the study is that modafinil promotes enhanced centrality, a measure of the importance of nodes within functional networks, of the bilateral primary visual (V1) cortex. EC analysis also revealed that modafinil-treated subjects show increased functional connectivity between the V1 and specific cerebellar (Crus I, Crus II, VIIIa lobule) and frontal (right inferior frontal sulcus and left middle frontal gyrus) regions. Present findings provide functional data supporting the hypothesis that modafinil can modulate the cortico-cerebellar connectivity of the aging brain.

## Introduction

In the last few years, psychotropic drugs, traditionally employed to treat a range of neuropsychiatric disorders, have also been evaluated with the aim to potentiate the cognitive performances of healthy individuals. The use of cognitive enhancing drugs (CEDs) is gaining growing attention (Dance, 2016) as these molecules may be of some help to maintain optimal brain functioning or even to compensate the subtle and subclinical deficits associated with brain aging or early-stage dementia. A rough classification of CEDs encompasses compounds that, directly or indirectly, influence cognition and behavior by acting on cholinergic, catecholaminergic, glutamatergic, histaminergic as well as glucocorticoid-dependent pathways (Fond et al., 2015).

Within the CED subgroup of compounds targeting dopaminergic and/or adrenergic neurotransmission, modafinil (trade name Provigil) stands out as a non-amphetamine-like wake-promoting drug that has been employed to treat excessive daytime sleepiness associated with narcolepsy, shift-work sleep disorders, obstructive sleep apnea, and cataplexy (Minzenberg and Carter, 2008). Off-label use of modafinil has also been clinically tested to treat cognitive dysfunctions often associated with the Attention Deficit/Hyperactivity Disorder (ADHD; Volkow et al., 2009), mood disorders (Goss et al., 2013), schizophrenia (Dawson et al., 2012), fatigue in multiple sclerosis (Kraft and Bowen, 2005), and Parkinson’s disease (Tyne et al., 2010).

Modafinil acts on subcortical structures, namely the thalamus, hypothalamus, and amygdala to reinforce physiological mechanisms involved in the activation and maintenance of wakefulness (Gerrard and Malcolm, 2007). Modafinil exerts its action by competitively binding to the dopamine transporter (DAT) as well as by inhibiting norepinephrine uptake, thereby producing an overall elevation of catecholamine levels and potentiation of adrenergic neurotransmission (Qu et al., 2008). Modafinil can also increase extracellular brain levels of serotonin, glutamate, histamine, and orexin as well as decrease the release of gamma-amino-butyric acid (Minzenberg and Carter, 2008).

Recent behavioral studies have indicated that modafinil can enhance cognitive performances in domains like attention, memory, executive functions, and increase alertness and response accuracy (Battleday and Brem, 2015). Furthermore, in preclinical settings, the drug has been shown to promote hippocampal neurogenesis (Brandt et al., 2014) and synaptic plasticity (Rao et al., 2007; Tsanov et al., 2010). Overall, this psychostimulant has been viewed as a CED with lower risks of inducing addiction and a favorable side effect profile (Malcolm et al., 2002).

Neuroimaging studies have helped to clarify modafinil effects on functional connectivity across brain network nodes (Esposito et al., 2013; Cera et al., 2014; Battleday and Brem, 2015). However, most of these functional Magnetic Resonance Imaging (fMRI) studies investigated modafinil or dopamine-related CED effects in young subjects while not much evidence is available on CED activity on the aging brain.

In this study, with fMRI, we assessed effects on functional connectivity (FC) evoked by the administration of a single dose (100 mg) of modafinil in a sample of healthy elderly individuals. Resting state fMRI (rs-fMRI) effects were also, in parallel, tested in an age-matched group undergoing placebo treatment.

## Materials and Methods

### Study Subjects

The study was conducted at the Institute of Advanced Biomedical Technologies (ITAB), a clinical research center of the Chieti-Pescara University, and involved twenty-four (16 females and 8 males) participants divided into two study groups matched for age, sex, and education (**Table 1**). We enrolled subjects (age range 57–75 years old) with comparable levels of education (11 ± 3.9 years). Study eligibility was determined after complete physical and neurological examinations by an expert neurologist. Moreover, participants were evaluated by careful and thorough radiological history to exclude unsuitability for MRI scanning. To determine the global cognitive status, we employed the Mini-Mental State Examination (MMSE- Folstein et al., 1975), a widely used test that allows evaluation of orientation, attention, memory, language, and visual-spatial skills. All the recruited participants had MMSE ≥ 27 scores. Subjects with past or current psychiatric disease and neurological deficiencies were excluded. None of the study participants suffered or was suffering from major illnesses or had a history of tobacco, alcohol, and/or psychoactive drug abuse. None of the volunteers was actively smoking at the time of the study. All the participants were consuming what is considered a regular intake of 1–2 Italian espresso per day. Participants were asked to avoid alcohol consumption 12 h before the beginning of the study and to maintain their usual levels of intake of caffeine.

**Table 1:** Demographic variables (gender, age, and years of formal education) of the total sample, modafinil group, and placebo group, separately.

Group	Number	Gender (males)	Age (means, years ± SD)	Education (means, years ± SD)
Modafinil group	12	12 (4)	66.5 ± 4.66	10.66 ± 4.0
Placebo group	12	12 (4)	66.08 ± 5.21	11.5 ± 4.07
Total sample	24	24 (8)	66.29 ± 4.8	11.08 ± 3.9

After providing written informed consent, study participants received, in a double-blind and randomized fashion, a single-dose of modafinil (modafinil group, *n* = 12) or a placebo pill identical to the drug pill (placebo group, *n* = 12). All subjects then underwent two fMRI scans, performed before and 3 h after drug (or placebo) administration, to achieve a plateau phase in drug levels in line with the compound pharmacokinetic profile (Robertson and Hellriegel, 2003). The study followed the tenets of the Declaration of Helsinki and was approved by the local Ethics Committee (PROT 2008/09 COET on 10.14.09).

### MRI Data Acquisition

Resting state-fMRI Blood oxygen level-dependent (BOLD) data were subdivided into two runs, lasting 4 min each, followed by T1- weighted multi-echo MPRAGE sequences for anatomical reference. The choice of two runs was driven to increase the overall reliability and quality of BOLD signals and accommodate the participant’s request to minimize the discomfort of long MRI acquisitions. This choice also significantly reduced the impact of artifacts due to movements. Long BOLD time-series, for each subject, maximized the statistical power when calculating Pearson’s correlation coefficient.

Subjects were asked to relax and fix a central point in the middle of a background screen. Upon rs-fMRI acquisitions, participants were instructed to stay still, keep their eyes open on a fixation cross, and do not think about anything in particular. All images were acquired with an eight-channel coil. Foam pads positioned around the head were added to reduce large involuntary movements. BOLD imaging was performed with a Philips Achieva 3T Scanner (Philips Medical Systems, Best, The Netherlands) and images acquired with T2^∗^-weighted echo planar imaging (EPI) sequence with the following protocol: TE (echo time) 35 ms, matrix size 64 × 64, FoV (field of view) 256 mm, in-plane voxel size 4 × 4 mm, flip angle 75°, slice thickness 4 mm and no gaps. 140 functional volumes of 30 axial slices were acquired per run with a volume TR (repetition time) of 1671 ms. A high-resolution structural volume was acquired at the end of the two rs-fMRI runs through a 3D magnetization prepared rapid gradient echo (MPRAGE) sequence employing the following parameters: sagittal, acquisition matrix = 256 × 256, FoV 256 mm, slice thickness 1 mm, no gaps, in-plane voxel size 1 mm × 1 mm, flip angle 12°, TR = 9.7 ms and TE = 4 ms.

### Data Processing and Statistical Analysis

fMRI data were pre-processed by applying corrections for head motion, removal of non-brain voxels and slice-timing corrections (performed using FSL: FMRIB’s Software Library) ^1^. Several sources of physiological variance were mitigated from each subject time-series. Six parameters of realignment and their derivatives, the first five eigenvectors of the PCA decomposition of the EPI time course, averaged over cerebrospinal fluid (CSF) and white matter (WM), were regressed out, following the CompCor approach for physiological noise removal (Behzadi et al., 2007). Head motion estimation parameters were used to derive frame-wise displacement (FD). Time points with high FD were replaced by a cubic-spline interpolation (Power et al., 2012). Data were then demeaned, de-trended and band-pass filtered in the frequency range of 0.01–0.1 Hz. A two-step registration process was performed for group analysis. fMRI data were transformed, linearly, from functional space to individual subject structural space using the FMRIB’s Linear Registration Tool (FLIRT) and then, non-linearly, to a standard space (Montreal Neurological Institute standard map – MNI 152) using Advanced Normalization Tools (ANTs; Penn Image Computing & Science Lab)^2^. Finally, data were spatially smoothed (5 mm × 5 mm × 5 mm full-width half-maximum Gaussian kernel). At the end of the pre-processing, immediately before the registration to the standard space, two fMRI datasets for each subject were registered one to the other and merged after temporal mean removal and variance normalization.

### Network Analysis

To investigate changes in functional brain network organization induced by drug administration, we modeled resting state FC as a complex network (Caldarelli, 2007). Network analysis investigates node centrality. However, alternative definitions are available to describe the concept. Eigenvector Centrality (EC) was chosen as a topological metric to disclose brain nodes that were functionally connected to other highly functionally connected nodes (Rubinov and Sporns, 2010). Estimation of the degree of centrality was indirectly included as a parameter to calculate the threshold based on Erdos–Renyi entropy.

A functional connection between two brain areas was considered as an undirected (i.e., without a specific orientation) and weighted (i.e., with a value associated with the connection) graph link. The weight used for each link is the correlation coefficient that is calculated taking into consideration the time-series associated with the two areas (Gili et al., 2013). R-squared correlation coefficients were considered as similarity indices to account for correlations that result from neural-mediated, temporally and spatially heterogeneous, hemodynamic mechanisms (Goelman et al., 2014). It should be taking into account that this approach may potentially lead to some loss of information. In fact, positive and negative correlations, when carrying the same absolute values, refer to the same amount of information regarding connectivity strength. As we evaluated undirected weighted graphs, the phase-shift between time-series is, therefore, lacking information on the positive or negative value of the correlation. For each subject, the square value of the N × N correlation matrix R was calculated (N being the number of voxels of the gray matter considered) and a threshold applied to ensure that the Erdos–Renyi entropy of the network (*S*) was equal to 2 across subjects (Hayasaka and Laurienti, 2010). *S* = 2 corresponded to a specific correlation value for each subject, thereby leading to the same amount of surviving matrix elements after threshold. This approach maximizes the consistency of results. The applied threshold is very conservative and takes into account a restricted (0.4–0.5) range of correlation coefficients that are associated with *S* = 2. The stability and robustness of functional brain networks as function of *S*, when large values (*S* = 1, 1.5, 2) are employed, has been previously shown to reliably occur in healthy subjects (Hayasaka and Laurienti, 2010; Gili et al., 2013) or stroke patients (Gili et al., 2016).

*S* was calculated as the ratio between the logarithm of the total number of nodes and the logarithm of the normalized degree of the network (Watts and Strogatz, 1998). A generalization of the EC was then calculated from the resulting matrix (Newman, 2004). EC assigns relative scores to all nodes in the network assuming that connections to highly scoring nodes (highly connected nodes) contribute more to each score than equal connections to low-scoring nodes (poorly connected nodes), thereby shifting the ranking procedure to a multi-level perspective. It must be added that EC is based on the eigenvector decomposition of similarity matrices. Since the existence of the largest eigenvalue of a matrix is guaranteed in case of a semidefinite positive symmetric matrix (Perron–Frobenius theorem), the choice of taking in account the square of correlation coefficients, which can assume both positive and negative values, is appropriate. EC has been proven to be robust, reliable, not computationally demanding, and, unlike other measures, able to capture aspects of centrality that encompass global features of the graph. Other centrality measures were not considered for either computational reasons (betweenness) or because of the lack of information added (laplacian, katz).

The resulting centrality maps were then transformed (van Albada and Robinson, 2007) to ensure that they obey to a Gaussian normal distribution as required for subsequent statistical testing. To examine the influence of modafinil on the group level statistics, paired *t*-tests were performed to identify areas in which centrality varied significantly with the neurophysiological state. The analysis was corrected for subject gender, including it as a nuisance variable. Changes of EC (Nichols and Holmes, 2002) were identified from a permutation-based non-parametric within-subject paired analysis (FSL randomize). This analysis modeled the interaction of the effect of treatment, namely baseline pre-treatment (B) and end of treatment (E), and the effect of condition, namely D (drug) and P (placebo). The interaction is described by the contrast (ED–BD) – (EP–BP). Positive and negative interaction effects were examined. The results were subjected to threshold-free cluster enhancement (Smith and Nichols, 2009) and family wise error (FWE) corrected for multiple comparisons by permutation testing using a significance level of *p* < 0.05.

### *r*^2^ ROI-Based Analysis

To elicit the origin of centrality changes, we investigated patterns of changes of brain voxels *r*^2^ with respect to one specific Region of Interest (ROI). EC reports the strength of voxel-to-voxel connections but also reflects and captures the global features of the graph (Caldarelli, 2007). Accurate EC estimation of a whole graph G = (V, E) can be nevertheless achieved by appropriate tuning of the weights of subsets of the total links (Nicosia et al., 2012). The only constraint of this approach is that N links must belong to a subset E′⊆E as such that, for every node i∈V, there is a link l ∈ E′ pointing to i (Nicosia et al., 2012). Since, in the present study, we have identified a change of EC in just one cluster, it is possible to proceed with the investigation of the dependence of this change by looking at changes in connectivity between the cluster and its connections. Structured in this way, the procedure does not produce loss of generality.

The primary visual cortex (V1) or Brodmann Area 17 (BA17) was chosen accordingly to the results of network analysis. BA17 ROI was defined in MNI 152 space from the preceding analysis. *R*^2^ maps for each subject were obtained by squaring the correlation coefficient calculated between the average time series from the ROI and all the voxels of the brain. The resulting images were combined in a not- parametric permutation inference by FSL randomize (Nichols and Holmes, 2002); within-subject paired *t*-tests were used to assess increase of connectivity as consequence of modafinil action, controlled for the placebo condition as described in the network analysis section. Changes were considered statistically significant at *p* < 0.001 voxel level uncorrected and *p* < 0.05 cluster level FWE corrected. Gender was included as a nuisance variable.

## Results

The main results of this study relate to centrality and functional network changes evoked by acute administration of modafinil.

### Centrality Changes Induced by Acute Administration of Modafinil: Effects on the Primary Visual Cortex

**FIGURE 1:**
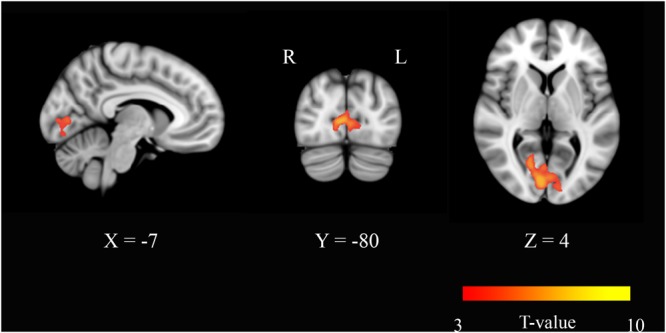
**Eigenvector centrality (EC) changes induced by acute administration of modafinil or placebo.** The image depicts maps of a paired *t*-tests obtained from the contrast (drug effect > drug baseline) > (placebo effect > placebo baseline) and considering the gender as a variable of not interest. A significant EC increase was observed in the primary visual cortex bilaterally (BA17) in the modafinil study group. Depicted clusters survived correction for multiple comparisons across space with FWE correction at *P* values < 0.05. Coordinates refer to MNI space.

Eigenvector centrality maps were calculated on networks composed of ∼30,000 gray matter voxels. The threshold was chosen by setting *S* = 2 in each connectivity matrix. This threshold conferred small-worldness to the networks and produced sparse matrices whose non-zero elements were 15% of the total entries. Paired *t*-tests were performed across the study group [(ED-BD) > (EP-BP)] to identify areas where EC varied significantly with the neurophysiological status. In the modafinil group, in the post-drug period, we found an increase of centrality that occurred bilaterally in the BA17 (**Figure 1**), thereby suggesting an increase of the FC of the visual cortex with other brain regions due to drug action. No significant changes were found in the opposite contrast [(BD-ED) > (BP-EP)].

### Functional Network Changes Induced by Acute Administration of Modafinil

To further investigate these modafinil-driven centrality changes, ROI-based FC analysis was performed using BA17 as resulted from the group statistics of EC maps. FC analysis revealed connectivity increase within the cerebellar Crus I, Crus II areas, and VIIIa lobule, the right inferior frontal sulcus (IFS), and the left middle frontal gyrus (MFG). Paired *t*-test of the pre- and post-drug imaging data showed increased connectivity of BA17 with these cerebellar and frontal lobe areas, thereby supporting a drug effect in the modulation of cortical and cerebellar connectivity.

**FIGURE 2:**
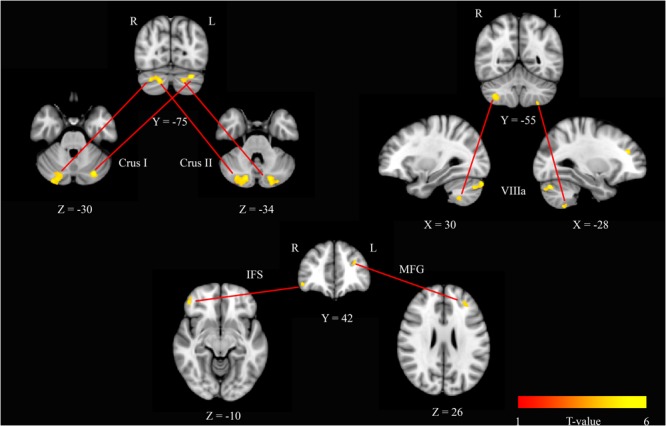
**Seed based *r*^2^ connectivity analysis of the BA17 region in the modafinil and placebo groups.** The image depicts maps of a paired *t*-tests calculated for the contrast (drug effect > drug baseline) > (placebo effect > placebo baseline) and considering the gender as a variable of not interest. Significant increases of *r*^2^ were observed in the left and right cerebellar Crus I, the left and right cerebellar Crus II, the left and right cerebellar VIIIa, the right IFS and the left MFG. Depicted clusters survived correction for multiple comparisons across space with FWE correction at *P* values < 0.05. Coordinates refer to MNI space.

The V1 showed a specific pattern of increased connectivity within the left [-29, -76, -30] and right [21, -79, -30] cerebellar Crus I, the left [-23, -84, -34] and right [17, -80, -34] cerebellar Crus II, the left [-27, -57, -56] and right [34, -53, -50] cerebellar VIIIa, the right IFS [44, 46, -8], and the left MFG [-29, 38, 23] (**Figure 2** and **Table 2**).

**Table 2:** Peak voxel coordinates of regions showing increased functional connectivity with the BA17 due to drug effect.

Brain region	Cluster size (vox)	Voxel (*x, y, z*)	Voxel (T)
Crus I of cerebellum (R)	2507	(21, -79, -30)	4.78
Crus II of cerebellum (R)		(17, -80, -34)	4.88
Crus I of cerebellum (L)	1251	(-29, -76, -30)	5.53
Crus II of cerebellum (L)		(-23, -84, -34)	4.64
Lobule VIIIa of cerebellum (R)	661	(34, -53, -50)	4.46
Lobule VIIIa of cerebellum (L)	545	(-27, -57, -56)	4.28
Inferior frontal sulcus (R)	460	(44, 46, -8)	4.43
Middle frontal gyrus (L)	450	(-29, 38, 23)	4.99

*Coordinates are reported in MNI space (mm). The voxel size was 1 mm × 1 mm × 1 mm and regions are grouped according to the cluster to which they belong. L, Left; R, right; BA, Brodmann area.*

## Discussion

In this study, we examined acute rs-fMRI effects on sub-regional FC evoked by the administration of a single dose of modafinil in a study group composed of healthy elderly subjects. Rs-fMRI is widely employed to investigate the activity of functional brain networks (Friston et al., 1993). Rs-fMRI is based on fluctuations of BOLD signals that temporally correlate with the spontaneous neuronal activity occurring in spatially distant brain regions that are not, necessarily, structurally related. Functional correlates of rs-fMRI are assumed to refer to self-referential thoughts, awareness of sensory inputs and dynamic environmental changes (D’Argembeau et al., 2005; He, 2013).

In the study, FC data were analyzed with a graph-based approach and algorithms set to highlight gray matter nodes based on their connectivity strength. This correlation-based FC analysis identifies and unravels, in a quantitative way, statistical associations between changes in BOLD signals occurring in cortical regions.

In the modafinil-treated group, we found, in the post-drug period, greater centrality in the bilateral primary visual cortex [BA17], the brain region highly specialized for processing visual information.

Functional connectivity analysis also revealed a drug-related involvement of the cerebellum. Modafinil administration was, in fact, found to be associated with increased connectivity between the V1 and the cerebellar Crus I, Crus II, and VIIIa lobule. Additional major FC changes were found in V1 connections with the right IFS and the left MFG of the frontal cortex.

These frontal areas are known to modulate attention levels and some core processes associated with executive functions, and, specifically, inhibitory control and working memory (WM). These functions depend on each other and co-activate frontal areas along with the posterior visual cortex to re-orient attention toward visual stimuli and also enhance cognitive efficiency.

Data on behavioral effects of modafinil administration in our study group are missing and, at this stage, we can only provide theoretical speculations for the functional correlates of the regional activations that we have found to be promoted by the drug.

A conceptual framework for the FC effects on the V1 may take into account that rs-fMRI study participants, even though scans are performed in “resting conditions,” are nevertheless asked to employ significant levels of visual alertness. Subjects are asked to keep their eyes open, look at a fixation cross, stay still, and retain these commands throughout the whole scanning period. Thus, to fulfill rs-fMRI requirements, subjects need to activate voluntary inhibition of movements, maintain visual attention as well as engage the WM.

Thus, within this framework, it is conceivable that the modafinil-driven increased FC we have found to occur between the V1 and some prefrontal areas reflects the active involvement of visual-, attention-, and WM-dependent networks that were set in motion by the rs-fMRI protocol and, possibly, boosted by the drug.

The V1 effects should be considered in relation to the main function of this brain region. The V1 is crucial to allow visual perception (Boly et al., 2007), a process that combines a set of “bottom-up” activities that are stimuli- and sensory-driven with a “top-down” information flow originating from the associative regions of higher order. The top-down process influences patterns of recognition, thereby favoring dynamic improving or updating of the sensorial information flow. Thus, the process favors the orientation of selective attention to relevant stimuli, thereby favoring a filtering activity that prevents WM overload (Gazzaley et al., 2005).

As for the modafinil-driven effects on the frontal cortex, several studies have indicated that the MFG and the neighboring inferior frontal cortex (IFC) act to filter out noise and irrelevant stimuli, thereby re-directing attention to achieve accurate task performance (Hopfinger et al., 2000; Corbetta and Shulman, 2002; Badre and Wagner, 2005). Moreover, the right IFS, a key node of IFC, has been associated with WM-related processes like the activation of inhibitory control (Aron et al., 2004) and selective attention (Dodds et al., 2011). These functions work to complement what described for the V1, thereby suggesting an overall scenario in which modafinil may act to improve interference control.

Our FC results also indicate a major effect exerted by modafinil on the cerebellum resting activity. The cerebellum is emerging as a crucial modulator of cognition as evidence indicates that cerebellar projections target multiple associative areas in the parietal and prefrontal cortex (Stoodley and Schmahmann, 2009).

Our rs-fMRI data indicate the occurrence of enhanced modafinil-driven FC between the V1 and the cerebellar Crus I and II. These areas are involved in modulating attention and spatial processing as well as WM encoding and maintenance (E et al., 2014). We also found increased FC between the V1 and the VIIIa lobule, a cerebellar region associated with sensorimotor processing. Previous results have indicated that prefrontal areas, Crus I/II, VIII cerebellar lobule are part of a cortico-cerebellar closed-loop (Salmi et al., 2010).

Of note, despite the large evidence in preclinical and clinical settings showing modafinil-driven FC effects on the thalamus, our EC analysis failed to report a great drug-related enhanced centrality for this region (Urbano et al., 2007; Minzenberg and Carter, 2008). Given that most of the studies have shown drug-related effects in young subjects, we can only speculate that the lack of major thalamic involvement that we found in our elderly sample may indicate lower sensitivity to modafinil of this subcortical region upon aging.

The net behavioral correlates of modafinil-driven modulation of network activities are largely unknown and likely to be complex. Preclinical evidence suggests potential beneficial effects. In theory, modafinil-driven FC changes may lead to enhanced experience-dependent synaptic plasticity and neurogenesis as well as behavioral and cognitive benefits (Minzenberg and Carter, 2008). The notion is supported by preclinical evidence showing that modafinil, through increased dopaminergic and noradrenergic transmission, promotes adult neurogenesis, especially within the hippocampus (Brandt et al., 2014). Furthermore, in preclinical models, modafinil has been shown to improve spatial learning and fear conditioning, inhibitory control, WM functioning, and sustained attention (Morgan et al., 2007; Shuman et al., 2009; Tsanov et al., 2010). Our studies provide some FC evidence that corroborates the hypothesis that modafinil may interfere with some of these cognitive domains.

## Conclusion

The potential therapeutic implication of modafinil use in the elderly can especially be envisioned when considering aging- or Alzheimer’s disease (AD)-dependent cognitive deficits.

Upon brain aging, cognitive deficiency can result from failure of inhibitory processes and the dysfunctional interplay between the WM and the attentional systems (Luis et al., 2015). This dysfunction can result in an overall reduced activity of filtering relevant information while increasing the production of noise overflow (Luis et al., 2015). A similar deficit in cognitive control has been found to occur in mild cognitive impairment (MCI) or early-stage dementia patients. Thus, on a speculative note, the modafinil-driven FC effects we are reporting to occur in areas that are controlling attention and inhibitory control may help to counteract deficits of the aging brain as well as in the brain of MCI or AD patients.

Interestingly, recent data indicate that AD-related neurodegeneration is present, beyond the cortex, also in the Crus I and II cerebellar regions (Guo et al., 2016). In that respect, it is conceivable that the modafinil FC effects we have found in these cerebellar areas may be of some help and exert a compensatory role.

There are some limitations of the present study. One limitation relates to the acute nature of our pharmacological intervention as chronic administration of modafinil may produce different activation patterns. An additional limit of the study concerns the lack of investigation of behavioral effects evoked by the acute administration of modafinil. Furthermore, in the age of precision medicine, further information is warranted on CED and modafinil activity in relation to factors such as genetic background, psychosocial features, as well as baseline levels of performance, a set of critical elements that can very differently shape drug responses in each individual.

## Ethics Statement

This study was carried out in accordance with the recommendations of the Ethics Committee for Biomedical Research of “G. d’Annunzio” University of Chieti and Pescara with written informed consent from all subjects. All subjects gave written informed consent in accordance with the Declaration of Helsinki. The protocol (PROT 2008/09 COET on 10.14.09) was approved by the Ethics Committee for Biomedical Research of “G. d’Annunzio” University.

## Author Contributions

SS conceived and designed the current study, supervised the data acquisition and analysis, and wrote the manuscript. MP supervised the data analysis and wrote the manuscript. TG performed fMRI data analysis and wrote the draft of the manuscript. LP, CC, GS supervised the data analysis and wrote the draft of the manuscript. All authors read and approved the final manuscript.

## Conflict of Interest Statement

The authors declare that the research was conducted in the absence of any commercial or financial relationships that could be construed as a potential conflict of interest.

## Footnotes

1 www.fmrib.ox.ac.uk/fsl

2 http://www.picsl.upenn.edu/ANTS/
